# Intravitreal therapy—success stories and challenges

**DOI:** 10.1007/s10354-024-01070-8

**Published:** 2025-03-03

**Authors:** Daniel Egger, Katharina A. Heger, Matthias Bolz, Max P. Brinkmann, Katharina Krepler, Pia Veronika Vecsei-Marlovits, Andreas Wedrich, Sebastian M. Waldstein

**Affiliations:** 1 Department of Ophthalmology, Landesklinikum Mistelbach-Gänserndorf, Mistelbach, Austria; 2 Karl Landsteiner University of Health Sciences, Krems, Austria; 3 Paracelsus Medical University Salzburg, Salzburg, Austria; 4 Department of Ophthalmology, Kepler University Clinic, Linz, Austria; 5 Department of Ophthalmology, Johannes Kepler University, Linz, Austria; 6 Department of Ophthalmology, Klinikum Klagenfurt, Klagenfurt, Austria; 7 Department of Ophthalmology, Universitätsklinikum Schleswig-Holstein, Lübeck, Germany; 8 Karl Landsteiner Institute for Retinal Research and Imaging, Vienna, Austria; 9 Department of Ophthalmology, Klinik Landstraße, Vienna, Austria; 10 Department of Ophthalmology, Klinik Hietzing, Vienna, Austria; 11 Karl Landsteiner Institute for Processoptimization and Quality Management in Cataract Surgery, Vienna, Austria; 12 Department of Ophthalmology, Medical University of Graz, Graz, Austria

**Keywords:** Anti-vascular endothelial growth factor therapy, Diabetic retinopathy, Intravitreal gene therapy, Intravitreal injections, Neovascular age-related macular degeneration, Anti-VEGF-Therapie, Diabetische Retinopathie, Intravitreale Gentherapie, Intravitreale Injektionen, Neovaskuläre altersbedingte Makuladegeneration

## Abstract

Intravitreal injections have revolutionized the treatment of various sight-threatening diseases of the posterior segment of the eye. Initially explored for treatment of bacterial endophthalmitis, intravitreal injections rapidly expanded to combat retinal vascular disease in particular. Especially anti-vascular endothelial growth factor agents have emerged as a cornerstone of intravitreal therapy, targeting neovascular age-related macular degeneration and diabetic macular edema as important examples. Advances continue, with novel therapies such as complement inhibitors now available as treatment for geographic atrophy secondary to non-neovascular age-related macular degeneration, offering hope for a previously untreatable condition. Pioneering approaches such as the port delivery system and intravitreal gene therapy aim to improve treatment efficacy while minimizing patient burden. Despite notable successes, challenges for intravitreal therapies persist, including ocular and systemic complications and high treatment burden. Future research endeavors aim to address these challenges and enhance treatment outcomes. This comprehensive review critically evaluates the efficacy, safety, and cost-effectiveness of intravitreal injections, delving into emerging trends and future directions.

## Abstract (de)

Intravitreale Injektionen haben die Behandlung verschiedener Augenerkrankungen revolutioniert, indem sie einen wirksamen Therapieansatz für Krankheiten des posterioren Augenabschnitts bieten. Anfangs für die Behandlung der bakteriellen Endophthalmitis erforscht, haben sich intravitreale Injektionen rasch in anderen Bereichen etabliert. Insbesondere haben sich Injektionen mit „anti-vascular endothelial growth factor“ als Eckpfeiler für die Therapie von Gefäßerkrankungen der Netzhaut behauptet. Diese Fortschritte setzen sich mit neuartigen Therapien wie Komplementinhibitoren fort, die nun für die geografische Atrophie bei nicht-neovaskulärer altersbedingter Makuladegeneration zur Verfügung stehen. Neue Therapieoptionen wie das Port-Delivery-System und intravitreale Gentherapien zielen darauf ab, die Wirksamkeit der Behandlungen zu verbessern und gleichzeitig die Belastung für die Patienten zu minimieren. Trotz bemerkenswerter Erfolge gibt es gleichermaßen wichtige Herausforderungen, wie beispielsweise okuläre und systemische Komplikationen sowie den hohen Behandlungsaufwand. Künftige Forschungsbestrebungen zielen darauf ab, diese Probleme zu bewältigen und die Behandlungsergebnisse zu verbessern. Im vorliegenden Übersichtsartikel werden die Wirksamkeit, Sicherheit und Kosteneffektivität intravitrealer Injektionen kritisch bewertet, und es wird auf neue Trends und zukünftige Entwicklungen eingegangen.

## Introduction

Intravitreal injections (IVI) are nowadays the most commonly performed ophthalmic procedure worldwide, with steadily rising numbers [1]. Few interventions in ophthalmology can claim to have had such a transformative impact on the landscape of ocular therapeutics as IVI, which provide a reliable and effective tool against various sight-threatening conditions.

Before the era of IVI, most diseases of the posterior segment of the eye were largely inaccessible to drug therapy. Intraocular infections, for example, were mostly treated by systemic antibiotics. Although there is evidence that parenteral or oral administration of antibiotics provides measurable intraocular drug levels, those levels were often found to be below the minimum concentration needed to effectively inhibit the pathogen [2]. This can be explained by the avascularity of the vitreous body and the highly effective blood–ocular barrier, which together considerably limit penetration of systemically administered drugs [3].

Only in the early 1980s did ophthalmologists start to explore the possibility of administering drugs directly into the vitreous cavity. At first, this approach was primarily used to treat bacterial endophthalmitis with injections of intravitreal antibiotics [4], which was at that time established as the standard of care by a landmark clinical trial, the Endophthalmitis Vitrectomy Study [5].

With the advent of the acquired immunodeficiency syndrome (AIDS) epidemic in the 1980s/90s, ophthalmologists were facing another challenge. The spread of the cytomegalovirus (CMV), the most frequent opportunistic infection in AIDS patients, frequently resulted in CMV retinitis, with devastating effects on the retina and vision if left untreated [6]. Systemic administration of ganciclovir had limited success, and both ganciclovir and the only available drug to treat human immunodeficiency virus (HIV) infection at that time, zidovudine, were myelosuppressive [3]. Facing this conundrum, ophthalmologists began to inject ganciclovir intravitreally, which led to superior clinical outcomes but carried the disadvantage of requiring weekly injections due to ganciclovir’s short half-life [7, 8]. To target this issue, a sustained-release implant for ganciclovir was developed and later approved by the United States Food and Drug Administration (FDA) in 1996, allowing ophthalmologists to treat CMV retinitis effectively with a reduced treatment burden for patients [9–11].

Thus, treating intraocular bacterial and viral infections effectively via IVI marked the beginning of their success story and a paradigm shift in the treatment of sight-threatening ocular diseases. Today, IVI of anti-vascular endothelial growth factor (anti-VEGF) agents dominate the landscape of intraocular therapeutics. They form the cornerstone of treatment for millions of patients with retinal vascular diseases such as neovascular age-related macular degeneration (nAMD), diabetic macular edema (DME), and macular edema following retinal vein occlusion (RVO) and have revolutionized the management of these conditions, offering significant improvements in vision preservation and quality of life.

However, IVI are not devoid of challenges and controversies. Issues such as the burden of treatment, risk of ocular and systemic complications, and socioeconomic implications underscore the need for ongoing research and innovation in this rapidly evolving field. In this comprehensive review, we examine the evidence supporting the efficacy, safety, and cost effectiveness of IVI, while also exploring emerging trends, future directions, and unanswered questions that warrant further investigation. By shedding light on the remarkable impact of IVI in ophthalmology, our goal is to provide insights that may inform clinical practice of the broader medical community.

## Success stories

### Anti-vascular endothelial growth factor agents

Today, injections with anti-VEGF for various indications such as nAMD, DME, RVO, myopic choroidal neovascularization, and proliferative diabetic retinopathy account for most IVI administered around the world. In the pre-anti-VEGF era, a then still-unknown factor had long been presumed to promote new vessel growth in hypoxic retinas. In 1994, Miller et al. identified this factor to be VEGF by inducing retinal ischemia through laser photocoagulation of retinal veins in monkeys, which eventually led to iris neovascularization. Vascular endothelial growth factor was then successfully isolated in the aqueous humor and levels changed proportionally to the severity of iris neovascularization. This suggested VEGF to be a retina-derived diffusible factor promoting angiogenesis and vascular permeability [12]. These findings were supported in a study by Aiello et al. during the same year, which found VEGF to be present in ocular fluid samples of patients with ischemic retinal diseases such as RVO and diabetic retinopathy [13].

It then took a decade to translate these basic scientific findings into clinically applicable medicine. The first anti-VEGF drug for the treatment of nAMD to be approved by the FDA in 2004 and the European Medicines Agency (EMA) in 2005 was pegaptanib (Macugen®, Eyetech Pharmaceuticals, Pfizer, New York, USA). This approval made pegaptanib the first anti-VEGF agent available for the treatment of ocular neovascularization [14]. Earlier the same year, bevacizumab (Avastin®, Genentech, Roche, California, USA), a humanized anti-VEGF antibody, received approval for treatment of colon cancer [15]. Soon after, ophthalmologists became interested in bevacizumab’s anti-VEGF properties and began successfully treating patients with nAMD by off-label administration of systemic and intravitreal bevacizumab [16, 17]. Bevacizumab remains the most widely used anti-VEGF agent around the world, despite its continuing off-label status.

**Fig. 1:**
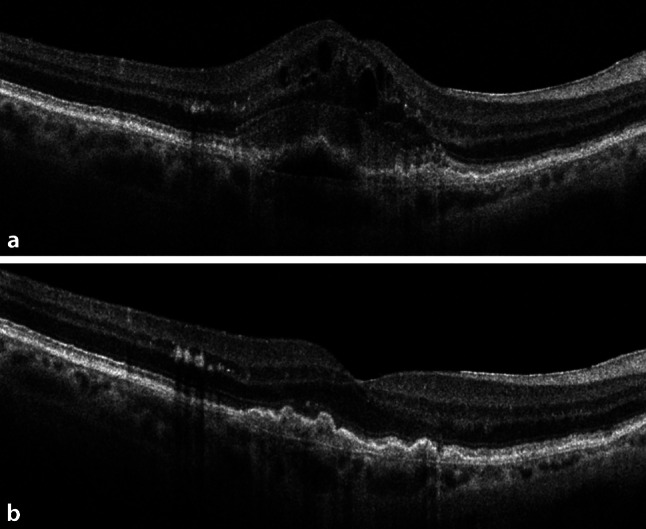
**a** Optical coherence tomography scan of a treatment-naïve patient with neovascular age-related macular degeneration showing macular edema with intraretinal fluid, subretinal hyperreflective material, and pigment epithelial detachment. **b** After one injection of bevacizumab (Avastin®, Genentech, Roche, California, USA) a dry macula is demonstrated

After approval of the anti-VEGF agent ranibizumab (Lucentis®, Genentech, Roche/Novartis, California, USA) by the FDA in 2006 and the EMA in 2007, this drug was soon considered the gold standard for nAMD treatment due to its increased efficacy compared to pegaptanib [18, 19]. During the 2010s, indications for ranibizumab were broadened to RVO and DME [20–23]. The efficacies of bevacizumab and ranibizumab treatments in nAMD were compared in a publicly funded randomized controlled trial and found to be equivalent, which further supported the continued use of bevacizumab in spite of the lack of formal approval (Fig. 1; [24]). During the same year, another anti-VEGF agent, aflibercept (Eylea®, Regeneron Pharmaceuticals, Bayer, New York, USA), was approved by the FDA and a year later by the EMA. With improved pharmacokinetics, i.e., a 100-fold increase in VEGF binding affinity, aflibercept 2 mg given every 2 months was found to be noninferior to ranibizumab every month [25]. Aflibercept also proven effective for the treatment of RVO and DME and allowed for higher intervals between injections to reduce patient burden [26, 27]. In 2024, aflibercept 8 mg (Eylea HD®, Regeneron Pharmaceuticals, Bayer, New York, USA) with a four-fold increase in dosage was approved by the FDA and the EMA for use in nAMD, DME, and RVO, with treatment intervals of up to 20 weeks [28, 29]. Brolucizumab (Beovu®, Novartis, Basel, Switzerland) was approved in 2019, but has lost popularity since then due to increased rates of intraocular inflammation (see below) [30, 31]. Faricimab (Vabysmo®, Genentech, Roche, California, USA), the first bispecific antibody targeting both VEGF and angiopoietin‑2, complements the approved anti-VEGF treatment options for nAMD, DME, and RVO, with approvals in 2022 and 2023 [32–34].

### Complement inhibitors

Ophthalmologists have been able to treat nAMD effectively with injections of anti-VEGF for almost two decades. However, for geographic atrophy (GA), the advanced disease stage of non-neovascular AMD characterized by subsequent atrophy of the outer retinal layers, there was no treatment available until recently. Progression of GA has been linked to the complement system, which is part of the innate immune response and comprises three different pathways. These pathways converge at factor C3 while also cascading through factor C5, which makes them promising targets for therapeutic intervention [35, 36]. The FDA approved pegcetacoplan (Syfovre®, Apellis, Massachusetts, USA), a C3 inhibitor, and avacincaptad pegol (Izervay®, Astellas Pharma, Illinois, USA), a C5 inhibitor, in 2023 for slowing progression of GA. This was based on the results of the four phase III trials, DERBY/OAKS [37] and GATHER1/2 [38, 39], respectively, which all showed slowing of GA progression but no effect on visual function.

Contrary to preceding approvals for nAMD, the FDA waived the requirement of improvement in functional endpoints such as visual acuity, scotoma size, or low luminance visual acuity and accepted slowing of GA expansion on fundus autofluorescence images as a primary anatomical endpoint for drug approval. These changed requirements as well as the only moderate effect on GA progression of pegcetacoplan and avacincaptad pegol (22% and 19% decreases of GA lesion growth with monthly injections over 24 months and 27% and 14% decreases with monthly injections over 12 months, respectively) raised questions concerning treatment burden, cost effectiveness, and patient benefit [40–42]. In an initial review, the EMA refused approval of pegcetacoplan [43] on the grounds of a lack of benefit for visual function. The EMA also referred to the increased rate of new-onset nAMD for patients receiving pegcetacoplan monthly or every other month versus patients receiving sham injections, which was reported as 13% and 6% versus 4% in DERBY and 8% and 11% versus 2% in OAKS, respectively, at 24 months [37]. Therefore, it might be beneficial to primarily offer this treatment to fast-progressing patients in order to prevent overarching treatment burden in the face of an on-average moderate efficacy. However, this is the first time that a treatment has shown any success in slowing of GA progression in clinical trials, which provides hope to millions of affected people around the world.

### Corticosteroids

In the early 2000s, the use of intravitreal steroids to treat retinal diseases such as DME, macular edema following RVO, uveitic macular edema, and pseudophakic cystoid macular edema (also known as Irvine–Gass syndrome) emerged. The rationale for using intravitreal steroids to treat retinal vascular diseases is the reduction of inflammation, which is often associated with abnormal cell proliferation, and the stabilization of the blood–retinal barrier to reduce vascular permeability [44]. The leading steroid to be administered intravitreally was triamcinolone and it provided excellent results, which at the time could already be monitored by optical coherence tomography (OCT), a noninvasive imaging technique that provides in vivo imaging of the retinal layers. Multiple trials for different indications that confirmed the effectiveness and safety of intravitreal triamcinolone were conducted in the following years and it accounted for most of the IVI administered in the early 2000s [45–48].

To further decrease the burden caused by frequent IVI, two sustained-release implants of corticosteroids were developed. Ozurdex® (Allergan, AbbVie, California, USA), which is a bioerodible implant releasing dexamethasone over a 3–6 month period, was shown to be effective in the treatment of DME [49], macular edema secondary to RVO [50], and posterior uveitis [51]. Iluvien® (Alimera Sciences, Georgia, USA), a non-bioerodible implant which releases a continuous amount of fluocinolone over a period of 2–3 years, was shown to be effective in the treatment of DME [52] and posterior uveitis [53].

## Challenges

### Ocular complications

**Fig. 2:**
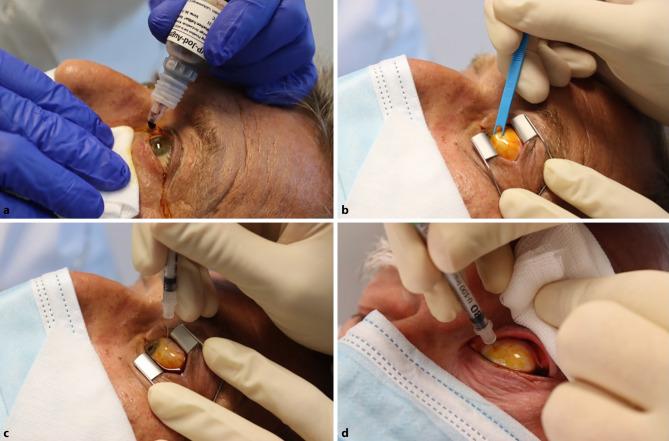
Process of administering an intravitreal injection. **a** Repeated ocular antisepsis with povidone-iodine. **b** After optional insertion of a lid speculum, a marker is used to identify the injection site. **c** After reapplication of povidone-iodine, the needle is introduced perpendicularly, and the agent is applied. **d** Intravitreal injection using manual lid retraction as an alternative to the lid speculum

Intravitreal injections carry a small but significant risk of adverse events (AE). Complications range from harmless subconjunctival hemorrhages and ocular discomfort to sight-threatening retinal detachment and endophthalmitis. To prevent these complications, standard operating procedures are maintained as follows: All involved parties should wear surgical masks to prevent contamination of the needle tip and operating field with oral bacterial flora. After instillation of topical anesthesia, repeated ocular antisepsis with povidone-iodine (Fig. 2a) and periocular antisepsis, a lid speculum may be introduced as per the physician’s discretion or lid retraction can be achieved manually (Fig. 2b, d). A marker can then be used to identify the injection site 3.5 mm (pseudophakic) or 4 mm (phakic) behind the limbus (Fig. 2b) and povidone-iodine is instilled again. The 30G needle is then introduced perpendicularly and the agent is applied (Fig. 2c, d). Povidone-iodine is instilled again, and a lubricant is applied to reduce foreign-body sensation. Proper patient education concerning postinterventional behavior, i.e., no rubbing of the eye, further reduces the risk of complications [1].

#### Minor complications

In a study evaluating patient-reported outcomes of 44,734 IVI, overall complication rates were low, with only 1.9% of patients reporting any AE. The most commonly reported mild complications were subconjunctival hemorrhage and irritation, accounting for 74% of all reported adverse events [54]. Silicone oil droplets in the vitreous originating from the silicone-oil-coated syringes used for IVI may cause symptomatic floaters in some patients and incidence ranges from 0.03% to 1.70% [55]. The use of silicone-oil-free syringes may be warranted to reduce the incidence of this minor complication [56].

#### Intraocular pressure elevation

Intravitreal corticosteroids can lead to increased intraocular pressure (IOP) due to higher aqueous humor outflow resistance in the trabecular meshwork [57, 58]. For dexamethasone, IOP elevation has been reported to occur in every third to every second patient [49, 59] and in up to two in three patients receiving triamcinolone [60, 61]. Acute IOP elevation after anti-VEGF injections is common and typically resolves within a few hours after the intervention. However, chronic IOP increases leading to glaucoma in patients receiving multiple IVI have been described in the literature, while the pathomechanism mostly remains unclear [62]. Therefore, IOP monitoring is warranted in patients receiving IVI over prolonged periods [63].

#### Cataract formation

If the needle compromises the posterior capsule during an IVI, a traumatic cataract may form and reduce visual acuity. This is a relatively rare AE but it may create an additional risk for complications in subsequent cataract surgery. Lens touch can be prevented by injecting 4 mm from the limbus in phakic and 3.5 mm from the limbus in pseudophakic eyes [64]. Previous IVI without compromising the posterior capsule still increases the risk of posterior capsular rupture (PCR) during cataract surgery by 4% per injection. However, a considerable number of prior IVI would have to be administered to significantly raise the risk of PCR, because of its low overall incidence of around 1% [65]. Additionally, cataract development is a well-documented complication following IVI of corticosteroids, possibly due to effects on proliferation and apoptosis of human lens epithelial cells [66, 67].

#### Vitreous hemorrhage

Vitreous hemorrhage has a reported incidence of 0.02% to 4.5% and often absorbs spontaneously [54, 68]. In case of fundus-obscuring vitreous hemorrhage, close follow-up with ocular ultrasound is warranted to exclude retinal detachment.

#### Retinal detachment

The overall incidence of retinal detachment after IVI is low, with rates of up to 0.6% [69]. Causes could be induction of posterior vitreous detachment or an inadequate site of injection. In patients with neovascularization, i.e., proliferative diabetic retinopathy, there is an increased risk (up to 5.2%) of tractional retinal detachment due to fibrotic processes after inhibition of VEGF [70].

#### Intraocular inflammation

Sterile intraocular inflammation (IOI) can include all segments of the eye, and its incidence is dependent on the administered drug. Brolucizumab has been found to have higher rates of IOI, particularly occlusive vasculitis, compared to aflibercept (4.4% vs 0.6%) [71].

#### Infectious endophthalmitis

Infectious endophthalmitis poses the most devastating complication of IVI, with potentially sight-threatening consequences. A same-day referral to an ophthalmologist and an immediate response with intravitreal antibiotics or pars plana vitrectomy is warranted if endophthalmitis is suspected in patients presenting with visual deterioration, profound redness, hypopyon, and ocular pain within days after an IVI [72]. The incidence ranges from 0.01% to 0.26% in most reports [73–75]. The most commonly isolated organisms causing endophthalmitis are coagulase-negative staphylococci, such as *Staphylococcus epidermidis*, which are part of the microbial flora of the conjunctiva, and *Streptococcus* species, especially viridans streptococci that form part of the oral flora [74, 76]. Therefore, the most important steps to prevent endophthalmitis after IVI are meticulous ocular antisepsis with povidone-iodine and the use of face masks for staff and patients. Draping, the use of sterile gloves, and a lid speculum may be considered, but have not been found to significantly reduce the risk of endophthalmitis [1].

#### Others

**Fig. 3:**
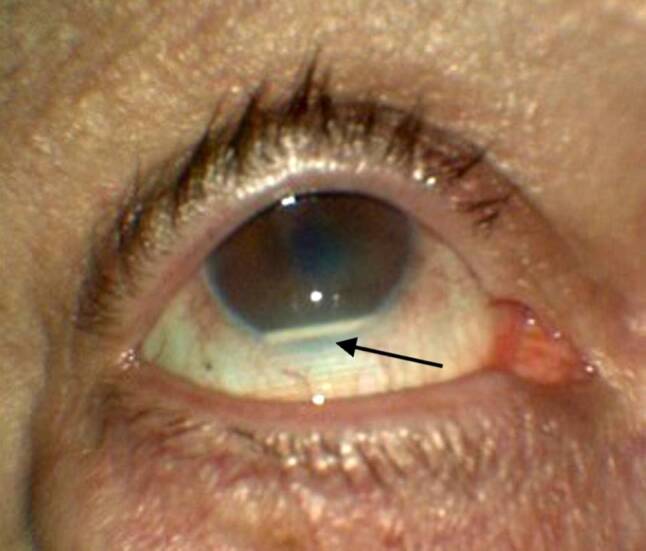
Migration of Ozurdex® (Allergan, AbbVie, California, USA) into the anterior chamber

Special care in patient selection has to be taken before administration of sustained-release corticosteroid implants, as factors such as zonular weakness in pseudophakic eyes may lead to migration of the implant into the anterior chamber and subsequent corneal decompensation and edema resulting in decreased visual acuity (Fig. 3; [77]).

### Systemic effects

Intravitreal injections are mostly administered in vulnerable patient populations such as the elderly or patients with diabetes and vascular diseases. Therefore, possible systemic effects of IVI must be considered. It has been shown that intravitreally administered anti-VEGF agents enter the bloodstream and reduce systemic VEGF levels [78, 79]. This also serves as an explanation for the “fellow-eye effect,” in which contralateral eyes that did not receive an anti-VEGF injection also showed anatomical improvements on OCT [80]. However, other systemic effects remain unclear to date, and conflicting results have been published. Anti-VEGF injections have been linked to gastrointestinal perforation and bleeding, hypertension, kidney disease, myocardial infarction, stroke, and thromboembolic events. However, in an overview of 21 systematic reviews and meta-analyses in 2018, Thulliez et al. found that anti-VEGF treatment did not increase the risk of systemic adverse events (syAE) when compared to controls. This finding was independent of treatment intervals. Only ranibizumab may be associated with a slightly higher risk of non-ocular systemic hemorrhage, such as subdural hematoma and gastrointestinal bleeding, especially in elderly patients with nAMD [81].

There also seems to be no difference in the syAE incidence among different anti-VEGF agents, as a study comparing the onset of syAE between patients treated with bevacizumab, ranibizumab, and aflibercept found no differences in the incidence of myocardial infarction, acute cerebrovascular disease, major bleeding, or hospitalization rates in 87,844 patients over an 11-year period [82]. Furthermore, a recent meta-analysis conducted in 2024 found no statistically relevant differences between the incidence of systemic arterial and venous thrombotic events in patients receiving ranibizumab vs. sham and between patients that received different anti-VEGF agents (aflibercept vs. bevacizumab vs. ranibizumab) for any indication [83]. In a study comparing intravitreal dexamethasone and anti-VEGF agents, again, no differences in incidence of syAE could be found [84].

By contrast, in a study in 2024, Zafar et al. found anti-VEGF injections in patients with type 2 diabetes to be independently associated with an increased likelihood (odds ratio 1.8) of syAEs, with the 5‑year cumulative incidence of any syAE being 37.0% in the anti-VEGF group compared to 18.4% in the non-injection group [85]. These findings contradict the results of the previously mentioned reports and underline the importance of prospective clinical trials with an a priori design to elucidate systemic effects of intravitreal treatment.

### Socioeconomic implications

It is estimated that over 7 million IVI per year are administered in the US, with the number projected to continue to increase further [3]. The cost of anti-VEGF agents, coupled with the need for repeated injections 6–12 times a year for a lifetime due to the indefinite nature of the disease, places a huge financial burden on healthcare systems. For example, from 2015 to 2019, the anti-VEGF agent aflibercept was the leading drug in overall Medicare Part B expenditure, which primarily accounts for outpatient/medical coverage. Costs for aflibercept, bevacizumab, and ranibizumab accounted for approximately 12% of all Medicare Part B drug expenditures, totaling over 4 billion USD in 2019 in comparison to 2.51 billion USD in 2014. This increase was largely driven by a trend toward more frequent use of aflibercept compared to other anti-VEGF agents. List prices for anti-VEGF agents range from 1850 USD and 1950 USD per dose for aflibercept and ranibizumab, respectively, to merely 60 USD on average per dose for bevacizumab [86].

**Fig. 4:**
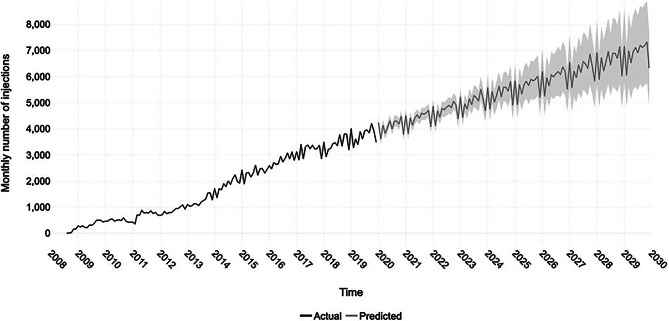
Monthly number of intravitreal injections (IVI) administered from 2008 to 2019 at Moorfield’s Eye Hospital London (black). Forecasted monthly IVI numbers until the end of 2029 with confidence intervals (grey) [82]. Licensed under CC BY 4.0 (https://creativecommons.org/licenses/by/4.0)

Likewise, numbers of IVI keep increasing in Europe. Moorfield’s Eye Hospital London, one of the largest eye hospitals in Europe, reported a nearly 11-fold surge from 2009 to 2019, with roughly 45,000 IVI administered in 2019 and a forecast of 83,000 injections in 2029 (see Fig. 4). Neovascular AMD was the leading indication and aflibercept, with 87%, was the most commonly administered drug [87]. In Austria, 212,000 IVI were administered in 2022, which represents a 4.4-fold increase compared to 2012. Numbers are predicted to rise to 252,000–346,000 IVI in 2030 [88]. While some retina specialists are mostly free to choose between different anti-VEGF agents, sometimes step therapy is mandated by health insurance, and hospital providers in publicly funded healthcare systems may mandate using a specific anti-VEGF drug, i.e., bevacizumab, as a first-line treatment to decrease medication costs [89, 90].

Aside from financial aspects, the treatment burden for patients cannot be overlooked. Repeated injections entail frequent visits to healthcare facilities, which can be physically taxing, especially for elderly and mobility-impaired patients, and reduce treatment adherence [91]. Other socioeconomic factors such as transportation costs, reduced quality of life, lost wages due to time off work, and caregiver burden remain. However, socioeconomic aspects also include benefits of anti-VEGF injections due to prevention of visual deterioration such as higher productivity, better quality of life, and decreases in long-term health costs such as home modifications and long-term care. Furthermore, effective treatment of retinal diseases helps patients to maintain their independence. This supports a healthier, more active aging population, which can contribute positively to the economy by reducing the demand for social and healthcare services.

The increasing workload of IVI also poses significant challenges for ophthalmologists and healthcare providers. The aging population and rising incidence of diabetes are driving up the prevalence of AMD and diabetic retinopathy. As new treatments for GA become available, the demand for IVI is expected to grow even further. Additionally, the anticipated retirement wave of ophthalmologists will exacerbate workforce shortage, placing increased pressure on the remaining professionals.

While newer drugs with longer intervals between injections have been approved recently, more research is needed to provide even more effective and longer-lasting treatment options to alleviate the burden on patients, caregivers, and healthcare providers.

## Future prospects

### Port delivery system

**Fig. 5:**
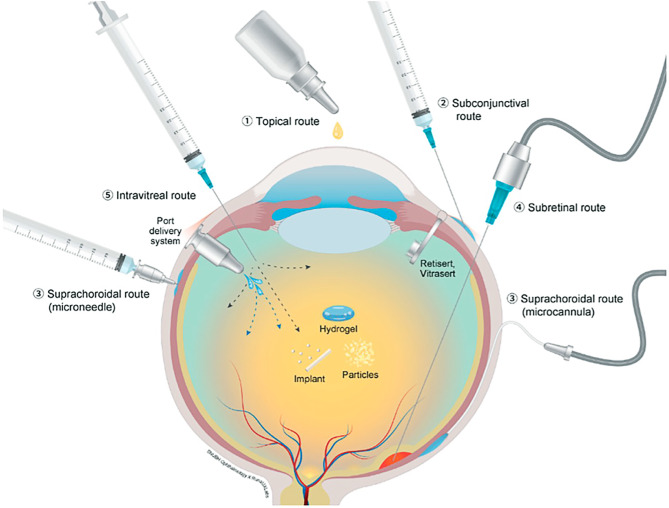
Various routes of ocular drug delivery [89]. Licensed under CC BY 4.0 (https://creativecommons.org/licenses/by/4.0)

Although IVI are highly effective, repeated injections place an enormous burden on patients, their relatives, and the healthcare system in general. To counter this issue, refillable implants with continuous drug release have long been desired. The most promising implant to date is the port delivery system (PDS) with ranibizumab called Susvimo® (Genentech, Roche, California, USA). It is a nondegradable and refillable implant, which is placed into the sclera at the pars plana by vitreoretinal surgery (Fig. 5). The concentration gradient between the port and the vitreous cavity results in passive diffusion of ranibizumab towards the vitreous. The pivotal Archway trial with 418 participants demonstrated noninferiority for patients implanted with a PDS (ranibizumab 100 mg/mL) and a refill exchange at week 24 compared to ranibizumab 0.5 mg every 4 weeks. Rescue therapy with ranibizumab 0.5 mg was possible but only needed by 1.6% of patients during the trial [92]. However, in October 2022, the PDS was recalled due to issues with septum dislodgement, which was recognized in 2.3% of patients after refilling the device in the Archway phase III trial and Portal extension study. In response to that, the device and refill needle have been remodeled and studies are currently ongoing [93].

### Intravitreal gene therapy

Gene therapy for retinal diseases offers a potentially long-lasting solution to conditions that currently require frequent and invasive interventions. To date, only an inherited retinal disease, namely Leber’s congenital amaurosis, can be treated effectively by gene augmentation with voretigene neparvovec (Luxturna®, Spark Therapeutics, Pennsylvania, USA) via adenovirus-associated virus vectors transporting the *RPE65* transgene into target cells [94]. However, Luxturna® (Spark Therapeutics, Pennsylvania, USA) has to be administered through subretinal rather than intravitreal injection (Fig. 4), which requires a surgical approach. An intravitreal delivery of therapeutic agents via IVI would provide a less invasive way to place the transgene.

Research efforts in intravitreal anti-VEGF gene therapy focus on viral vectors that safely and efficiently deliver genes encoding anti-VEGF proteins directly to retinal cells. These vectors are engineered to carry genetic instructions that enable the continuous production of anti-VEGF agents, potentially reducing or eliminating the need for repeated IVI. However, there are certain limitations to intravitreal gene therapy that have to be overcome first, such as dilution of the vector, the distance the vector has to surpass, and the host’s immune response [95, 96].

One of the most promising candidates is ixoberogene soroparvovec (ixo-vec®, Adverum Biotechnologies, Delaware, USA), a single-dose gene therapy encoding for the anti-VEGF protein aflibercept that can be administered intravitreally. It showed favorable results in a phase I trial, improving anatomical outcomes in nAMD and reducing the need for anti-VEGF injections [97]. Another candidate for anti-VEGF gene therapy is RGX-314, potentially providing continuous VEGF suppression by expressing an anti-VEGF‑A antigen-binding fragment. It also demonstrated promising results in a phase I/IIa trial but has to be administered via subretinal injection [98]. Although still under development, intravitreal gene therapy holds significant potential to revolutionize the treatment of retinal diseases currently managed with anti-VEGF injections. The encouraging results from early clinical trials indicate a future where retinal diseases can be managed more effectively.

## Conclusion

Tremendous advances have been made in the field of ophthalmology during recent years. A number of new intravitreal drugs to treat vascular retinal diseases have been approved and are already widely used in clinical practice. For the first time ever, a drug to treat GA following advanced “dry” non-neovascular AMD has been made available. Most of the diseases that ophthalmologists treat with IVI are of a chronic nature. This places an enormous burden on all parties involved—patients, caregivers, clinicians, and healthcare providers. Treatment burden and adverse events endanger treatment adherence. Research endeavors have to continue at the same pace to deliver even better and longer-lasting therapies, with the ultimate goal of providing best treatment outcomes with minimal burden.
